# Prioritizing Approaches to Engage Community Members and Build Trust in Biobanks: A Survey of Attitudes and Opinions of Adults within Outpatient Practices at the University of Maryland

**DOI:** 10.3390/jpm5030264

**Published:** 2015-07-28

**Authors:** Casey Lynnette Overby, Kristin A. Maloney, Tameka DeShawn Alestock, Justin Chavez, David Berman, Reem Maged Sharaf, Tom Fitzgerald, Eun-Young Kim, Kathleen Palmer, Alan R. Shuldiner, Braxton D. Mitchell

**Affiliations:** 1 Program in Personalized & Genomic Medicine, Department of Medicine, University of Maryland School of Medicine, Baltimore, MD 21201, USA; E-Mails: kmaloney1@medicine.umaryland.edu (K.A.M.); talestoc@medicine.umaryland.edu (T.D.A.); juscha1@umbc.edu (J.C.); david.berman@kcl.ac.uk (D.B.); rsharaf@medicine.umaryland.edu (R.M.S.); tfitzger@medicine.umaryland.edu (T.F.); eykim@inje.ac.kr (E.-Y.K.); kpalmer@medicine.umaryland.edu (K.P.); ashuldin@medicine.umaryland.edu (A.R.S.); bmitchel@medicine.umaryland.edu (B.D.M.); 2 Center for Health-related Informatics and Bioimaging, University of Maryland, Baltimore, MD 21201, USA; 3 University of Maryland, Baltimore County, Baltimore, MD 21250, USA; 4 King’s College London, London WC2R 2LS, UK; 5 Department of Clinical Pharmacology, Inje University Busan Paik Hospital, Busan 614-735, Korea; 6 Geriatric Research and Education Clinical Center, Veterans Affairs Maryland Health Care System, Baltimore, MD 21201, USA

**Keywords:** biobank, biorepository, research participation, public opinion, preferences, trust, data sharing, survey

## Abstract

*Background*: Achieving high participation of communities representative of all sub-populations is needed in order to ensure broad applicability of biobank study findings. This study aimed to understand potentially mutable attitudes and opinions commonly correlated with biobank participation in order to inform approaches to promote participation in biobanks. *Methods*: Adults from two University of Maryland (UMD) Faculty Physicians, Inc. outpatient practices were invited to watch a video and complete a survey about a new biobank initiative. We used: Chi-square to assess the relationship between willingness to join the biobank and participant characteristics, other potentially mutable attitudes and opinions, and trust in the UMD. We also used *t*-test to assess the relationship with trust in medical research. We also prioritize proposed actions to improve attitudes and opinions about joining biobanks according to perceived responsiveness. *Results*: 169 participants completed the study, 51% of whom indicated a willingness to join the biobank. Willingness to join the biobank was not associated with age, gender, race, or education but was associated with respondent comfort sharing samples and clinical information, concerns related to confidentiality, potential for misuse of information, trust in UMD, and perceived health benefit. In ranked order, potential actions we surveyed that might alleviate some of these concerns include: increase chances to learn more about the biobank, increase opportunities to be updated, striving to put community concerns first, including involving community members as leaders of biobank research, and involving community members in decision making. *Conclusions*: This study identified several attitudes and opinions that influence decisions to join a biobank, including many concerns that could potentially be addressed by engaging community members. We also demonstrate our method of prioritizing ways to improve attitudes and opinions about joining a biobank according to perceived responsiveness.

## 1. Introduction

Population-based biobanks are collections of donated bio-specimens such as tissue and blood samples that may also include donor-specific information, such as demographic and clinical information and genotypic data that can be stored for research purposes. Population-based biobanks are important resources that are used broadly for conducting large-scale health and/or genomic research. Biobanks hold promise for benefiting population health through facilitating investigations of disease incidence, risk of disease recurrence, and optimal therapies for both rare and common diseases.

In the US alone, there are over 600 collections of clinically annotated biological specimens, commonly called biobanks. Two-thirds of these biobanks were established within the past decade [1]. Biobanks can be exclusive to a particular medical condition, cater to a particular population, or both. Some examples include: the NIH National Cancer Institute’s Cooperative Human Tissue Network [2], which stores tumor samples and prospective clinical information; the EuroBioBank [3], a collection of samples and clinical information on individuals with rare disorders from multiple countries across Europe; the Wellcome Trust’s UK Biobank [4], which currently holds biological samples and health information on over 500,000 people; the Million Veteran Project, a collection of samples and health information of Veterans [5]; and the eMERGE (electronic Medical Records and Genomics) Network [6,7], an NIH National Human Genome Research Institute-supported study that is examining the utility of biobanks linked to electronic medical record systems at eight institutions. In addition to eMERGE Network participants, there are many other individual academic institutions and health systems that have developed biobanks for a broad range of biomedical research. Already, these biobanks have contributed to the identification of hundreds of loci for common diseases such as type 2 diabetes [8,9], cardiovascular disease [10,11], neurological [12,13], and psychiatric diseases [14,15]. Many biobanks also allow clinical information to be updated over time. Such access to clinical information allows scientists to perform longitudinal studies that measure factors influencing the progression, recurrence, and effectiveness of medications for treatment of disease [16].

While it is clear that biobanks hold promise for benefiting population health, achieving high participation of communities representative of all sub-populations is needed in order to ensure broad applicability of findings. Several studies, however, have identified potential barriers to participating in a biobank, including those surrounding privacy and confidentiality, the uncertainty of benefits they will see from participation, trust of the institutions and researchers running the biobank, and potential impact on identifiable racial and ethnic groups [17,18,19,20,21]. For example, in a survey of over 4000 US adults, 90% reported concerns about privacy, noting a fear of data being used against them. Nevertheless, 60% of respondents to this survey indicated they would still join in a biobank [18]. Privacy was also a major theme that arose in focus groups on genetic research, with the fear of discrimination based on genetic information being described as a barrier to biobank participation [21].

The goal of our study was to assess respondents’ views about biobanks and their potential willingness to donate a sample and clinical information to a new University of Maryland (UMD) biobank. We asked adults waiting in University of Maryland Faculty Physicians, Inc. outpatient practices to watch a brief informational video on biobanks and then to complete a short survey about their willingness to join the planned biobank. Our survey included questions about respondents’ comfort with donating samples and clinical information, change in willingness to donate with opportunities for community engagement, common concerns about donating, perceived benefits of donating, trust in medical research and UMD, and participant demographics and social characteristics. Data were analyzed to identify potentially mutable attitudes and opinions that are correlated with willingness to participate in a biobank. This knowledge will help biobank organizers to develop biobank policies and educational approaches that will help ameliorate concerns and encourage widespread community participation.

## 2. Materials and Methods

### 2.1. Informational Video and Survey Instrument Design

Our team developed all study materials, including an informational video and survey instrument. The video provided an overview of the planned University of Maryland biobank (UMBiobank) initiative and a brief introduction to this study (See Box 1 for questions covered by the video). The video described the planned UMBiobank as a collection of residual samples from UMD patients. A professional cameraman from the Office of Public Affairs of the University of Maryland School of Medicine recorded the 6-min video.

After reviewing surveys from other studies [19,22,23,24,25] and discussions among our research team, we created a 37-question survey. The survey consisted of seven main sections (See Appendix 1 for final survey):

(1) two-items: Video and primary question (“Do you feel like you understand what you just heard?” and “Do you think you would be willing to join the UMBiobank?”);
(2) eight-items: Comfort with sharing samples and clinical information (Example, “Please show how comfortable you are with the UMBiobank using your samples and different kinds of information: your blood samples”);
(3) five-items: Perceived responsiveness to approaches to engage community members and build trust in biobanks (Example, “Please show how your willingness to join the UMBiobank would change if: Members of my community have a role in making decisions about the biobank”);
(4) five-items: Common concerns about joining a biobank (Example, “Some people may have concerns about participating in a biobank. Please indicate your level of concern with the following: Researchers having my samples and information”);
(5) four-items: Perceived health benefits to joining a biobank (Example, “Please show if you agree or disagree with the following statements: It is important that my blood sample be used in research that could improve my own health”);
(6) four-items: Trust in medical research and the University of Maryland (Example, “Please show if you agree or disagree with the following statements: Medical researchers care only about what is best for each patient”; and 1-item: Trust in the University of Maryland (Example, “Would you want the University of Maryland deciding how your blood samples and clinical information are used in research?”); and
(7) eight-items: Demographic and social participant characteristics (Example, “What is your gender?”).

Our team included experts in genetic health communication to ensure that our video script and survey questions were appropriate for our patient population. With the exception of four questions, we also limited all question response options to two or three choices.

Box 1 **Questions covered in informational video on the University of Maryland biobank (UMBiobank) initiative.**

- What is a biobank?
- How are samples included in a biobank?
- Why is information from medical records needed with biobank samples?
- Will the privacy of individuals who agree to be in the biobank be protected?
- Who can use the biobank samples?
- Can people choose how their samples and information are used?
- Will results of research done on biobank samples be returned to individual participants?
- How long will biobank samples and information be used?
- Can participants who agree to be in the Biobank biobank change their mind?
- Will choosing to be in the biobank influence the care a patient receives at University of Maryland?

### 2.2. Sample and Recruitment Strategy

Between July and September 2014, research team members approached adults waiting in two University of Maryland Faculty Physicians, Inc. (FPI, Baltimore, MD, USA) outpatient practices (Family Medicine (FM) and General Internal Medicine (GIM)). These patients tend to be primarily residents of surrounding Baltimore neighborhoods, with University employees making up a small proportion. Potential participants were asked if they were willing to use an iPad the team member provided, to watch a short informational video about the UMBiobank initiative and to answer an anonymous questionnaire for research purposes. Participants were specifically informed that they were being asked to share attitudes and opinions about biobanking based on the information provided in the video and not for enrollment in the UMBiobank. Disposable headphones were provided to study participants for privacy purposes. Research data was collected and stored electronically using the online survey platform, Qualtrics (Qualtrics, LLC, Provo, UT, USA). The study was judged by the University of Maryland Institutional Review Board (IRB) as imposing only minimal risks on participants and was determined to be exempt from IRB review (HP-00059930).

### 2.3. Statistical Analyses

Survey respondents provided their age, gender, and preferred racial/ethnic identification (Black or African American, Non-Black or African American, and Prefer not to say). The 4-items about trust in medical research (see Appendix 1, Questions 26–29) were combined to create a trust in medical research score as previously described [25]. We initially estimated the proportion of respondents expressing willingness to join in a biobank by age, gender, racial designation, education, and experiences (with having children, with previous donation, and with having sick relatives). We then evaluated factors associated with willingness to join, including comfort with donating samples and clinical information, change in willingness to donate with opportunities for community engagement, common concerns about donating, perceived benefits of donating, trust in medical research and trust in UMD.

We evaluated the relationship of willingness to join the UMBiobank with a number of factors, including participant characteristics, trust in the University of Maryland, level of comfort in sharing samples and data, perception of health benefits of biobanks, and a variety of perceived concerns about biobanks. Chi-square tests and t-tests were used to compare characteristics between subjects willing to participate in a future biobank *vs.* those not willing to participate or unsure about participating. All statistical analyses were performed using Stata 13.1 (StataCorp LP, College Station, TX, USA).

## 3. Results

A total of 576 individuals were approached in the two outpatient practices and invited to participate. Of those approached, 294 agreed to participate, for a 51% response rate. Of those agreeing to participate, 169 (57%) were able to complete both the video and the survey before being called back for their appointment. Data analyses were performed on surveys completed from these 169 subjects.

The social and demographic characteristics of those completing the survey are provided in Table 1. Overall, most of the survey respondents were female (67%), non-black (60%), and well-educated (66% having completed at least some college, including receiving a bachelor’s and/or graduate or professional degree). Seventy percent had children, and 67% reported a 1st degree relative who had been affected by a major illness. Survey respondents were distributed relatively evenly across age distributions and nearly one-half reported that they had previously donated blood.

In response to the question “Do you feel like you understand what you just heard?” nearly all respondents (96%) indicated they felt as though they understood the video. Approximately one-half (51%) of respondents indicated that they would be willing to join the UMBiobank, with 25% reporting that they were unsure and 24% reporting that they would not be willing to join. Willingness to join the UMBiobank was not associated with age, gender, race, or education levels, nor was it associated with having children, history of donating blood, nor having first degree relatives with a major illness (see Table 1).

**Table 1:** Percent of survey completers (out of 169 total) willing to join the UMBiobank, according to demographic and social characteristics.

Demographic and Social Characteristics	Number of Participants, N (% of 169)	% Willing to Join ^§^
**Age**		
18–29	22.4	54.1
30–44	26.1	48.8
45–59	32.7	50.0
>60	18.8	67.7
**Gender**		
Male	31.5	47.2
Female	66.7	58.0
Prefer not to say ^♯^	1.8	0.0
**Race**		
Black or African American	37.1	45.2
Non-Black or African American	59.9	59.0
Prefer not to say ^♯^	3.0	60.0
**Education**		
<High School ^♯^	3.0	0.0
High School or GED	31.3	50.0
Some College	33.1	50.9
Bachelors	18.1	70.0
Graduate or Professional	14.5	58.3
**Children**		
Yes	69.7	56.5
No	30.3	46.0
**Donated before**		
Yes	45.5	57.3
No	50.3	48.2
Not Accepted ^♯^	4.2	71.4
**1st degree relative affected by a major illness**		
Yes	66.7	55.5
No	24.8	53.7
Unsure ^♯^	8.5	35.7

^♯^ Removed from *Χ*^2^ analysis. **^§^** None of the demographic or social characteristics were associated with willingness to join the UMBiobank (*p* ≥ 0.09 for all).

In terms of trust, 59% of survey respondents indicated that they would want the University of Maryland deciding how their blood samples and clinical information are used in research. Respondents who expressed a trust in the University of Maryland were significantly more likely to report that they would join the UMBiobank than those who did not, and trust in medical researcher was a predictor of willingness to join the UMBiobank (*p* = 0.0004). Among those indicating trust in UMD, 74% expressed a willingness to join the UMBiobank, compared to 21% among those who were unsure or did not trust UMD.

### 3.1. Comfort with Sharing Samples and Clinical Information

Given the background survey participants are provided in the informational video regarding “Who can use the biobank samples?” comfort with the UMBiobank using samples and clinical information, is referred to as *sharing* samples and clinical information. Individuals expressing a willingness to join a biobank were far more comfortable with sharing blood samples and data (92%–94% expressing comfort sharing) compared to subjects who were not willing to participate or unsure about participating, for whom only 28%–55% expressed comfort in sharing samples or data (Table 2). Moreover, as indicated in Table 2, only 28.6% and 33.3% of subjects expressed comfort with sharing blood samples and genetic results, respectively, while 35%–41% expressed comfort in sharing clinical information, and 55% expressed comfort in sharing basic demographic information.

**Table 2:** Willingness to join according to comfort sharing samples and clinical information.

Comfort with Sharing Samples and Clinical Information^§^	Comfortable with Sharing among Those Willing to Join (*n* = 90)	Comfortable with Sharing among Those Unsure or Unwilling to join (*n* = 78)
Your blood samples.	94.4%	28.6%
Your age.	94.4%	55.1%
Your gender.	94.4%	55.1%
Your ethnic group.	92.2%	55.1%
Your previous illnesses or diagnoses.	92.2%	41.0%
Your test results (e.g., any lab results, X-rays).	92.2%	34.6%
Your previous treatments (e.g., medications).	93.3%	39.7%
Your genetic information or genetic test results.	93.3%	33.3%

**^§^** *p* ≤ 0.001 for all comparisons.

### 3.2. Common Concerns about Joining Biobanks

We asked all survey respondents to indicate their level of concern with several issues cited in the literature as being barriers to participation in biobanks. Among these potential barriers, 68% of respondents indicated they had concerns about privacy and 55% indicated they had concerns about data being used against them. Less than half of respondents were concerned about researchers having their data (36%), being used as a guinea pig (31%), and discrimination (48%). Common concerns were strongly associated with willingness to join the UMBiobank, with 19%–58% concerned among those indicating a willingness to join the biobank compared to 52%–78% among those indicating they were unsure or unwilling to join (*p* ≤ 0.001 for all, Table 3). Of the 134 respondents indicating they had at least one common concern about participating in the UMBiobank only thirty-two (24%) indicated they would not be willing to join the biobank. Sixty-three individuals (47%) indicated they would be willing to join and thirty-nine individuals (29%) were unsure about joining.

**Table 3:** Willingness to join according to common concerns about joining biobanks.

Concerns about Participating in the UMBiobank	Concerned among Those Willing to Join (*n* = 90)	Concerned among Those Unsure or Unwilling to Join (*n* = 78)	*P*-Value (by Chi-sq Test)
Researchers having my samples and information.	18.9%	51.9%	*p* ≤ 0.001 *
Keeping my information private.	57.8%	77.9%	*p* ≤ 0.01 *
Information stored in the biobank being used against me.	42.2%	67.5%	*p* ≤ 0.001 *
Feeling like a guinea pig.	21.1%	41.6%	*p* ≤ 0.01 *
Information stored in the biobank being used to discriminate against people by race or ethnicity.	36.7%	57.1%	*p* ≤ 0.01 *

* *p* < 0.05.

### 3.3. Perceived Health Benefits to Joining Biobanks

The majority of respondents agreed it was important to them that their blood sample be used in research that could improve their health (64%), improve the health of people they love (70%), improve the health of others of the same race or ethnicity (68%), and improve the health of others in general (73%). Some perceived health benefits were strongly associated with a willingness to join the UMBiobank. For example, agreement in the importance of improving the health of others of the same race or ethnicity and the health of others in general was higher among those willing to join compared to those who were unsure or unwilling to join (76% and 82% *vs.* 54% and 61% respectively, *p* ≤ 0.01 for both). We did not detect any differences for other surveyed health benefits (see Table 4). 140 out of 168 respondents agreed in the importance of some health benefit. Of those, the majority (60%) indicated they were willing to join, 26% indicated they were unsure, and 14% indicated they would not be willing to join the biobank.

**Table 4:** Willingness to join according to perceived health benefit.

Important My Blood Sample be Used in Research…	Agreement in Health Benefit among Those Willing to Join (*n* = 90)	Agreement in Health Benefit among Those Unsure or Unwilling to Join (*n* = 78)	*p*-value (by Chi-sq test)
…that could improve my own health.	70.0%	57.7%	*p* = 0.097
…that will not affect my own health, but could improve the health of people I love.	75.6%	64.1%	*p* = 0.105
…that will not affect my own health, or the health of people I love, but could improve the health of others of the same race or ethnicity.	75.6%	53.8%	*p* ≤ 0.01 *
…that will not affect my own health, or the health of people I love, but could improve the health of others in general.	82.2%	61.5%	*p* ≤ 0.01 *

* *p* < 0.05.

### 3.4. Perceived Responsiveness to Approaches to Engage Community Members and Build Trust in Biobanks

Across all respondents, the majority indicated that they would be more willing to join the UMBiobank if the biobank was more responsive to the community. For example, 39% of respondents said they would be more willing to participate in the biobank if the community played a role in making decisions about the biobank, 51% would be more willing to participate if there were greater chances to be updated regularly about the biobank, 57% would be more willing to participate if there were more chances to learn more about the biobank, 46% would be more willing to participate if community concerns were placed first, and 38% would be more willing to participate if members of their community are leading biobank research. Across all approaches, 50%–70% of respondents indicated that they would be more willing to join the UMBiobank, 30%–68% among those unsure about joining, and 12%–23% among those unwilling to join (see Table 5).

**Table 5:** Willingness to join according to perceived responsiveness to approaches to engage the community and to build trust in biobanks.

Possible Interventions to Change Willingness to Join the UMBiobank	More Willing among Those Willing to Join (*n* = 90)	More Willing among Those Unsure about Joining (*n* = 44)	More Willing among Those Unwilling to Join (*n* = 34)
Members of my community have a role in making decisions about the biobank.	53.3%	29.5%	17.6%
There are chances to be updated regularly about the biobank (e.g., press releases, website updates).	64.4%	50.0%	23.5%
There are chances to learn more about the biobank (e.g., educational material).	70.0%	68.2%	23.5%
Concerns of my community are put first.	61.1%	40.9%	20.6%
Members of my community are leading biobank research.	50.0%	36.4%	11.8%

## 4. Discussion

In order to promote high community participation, biobank education initiatives, sample collection processes and data collection processes may be tailored to local contexts. In this study, we did not detect any differences in willingness to join in the biobank among the surveyed social and demographic characteristics (Table 1). It will still, however, be worth investigating whether there are differences in actual sample donation given that assessing hypothetical willingness to join in biobanks often do not provide an adequate estimate of actual participation rates. One survey study conducted in Baltimore, MD, for example, found there were differences in actual willingness to participate in genetic research by ethnic group [26]. Another study found that prior blood donation was a predictor of approval of genetic research [27]. We therefore focus our discussion on potentially mutable factors that could influence the decision of our patient population to join the biobank (Table 2, Table 3 and Table 4), and potential approaches to change willingness to join (Table 5). In the following sections, we explore two broad categories of mutable factors, public attitudes and desired benefit, in the context of proposed approaches. We also explore ways in which our findings regarding reasons to enroll in biobanks support findings from other studies with actual biobank participants’.

### 4.1. Public Attitudes toward Participating in a Biobank

Public attitudes we surveyed included (a) comfort sharing samples and information, (b) common concerns about biobanks, (c) trust in the University of Maryland, and (d) trust in medical research. Not surprisingly, we found that respondents who were comfortable with sharing blood and other information were more willing to join the UMBiobank. Interestingly, respondents who were unsure or unwilling to join the UMBiobank were more skeptical about contributing their blood samples and genetic results than other clinical data (see Table 2). Respondents who expressed common concerns were less willing to join the UMBiobank (see Table 3). Discomfort with sharing blood samples may be in part due to fear of specific biospecimen extraction procedures (e.g., fear of pain, seeing blood, or problems associated with blood collection). Regarding genetic results, public attitudes may be influenced by ideas that genetic data are different and riskier than other forms of health data (*i.e.*, genetic exceptionalism) [28]. More generally, discomfort with sharing information and common concerns were apparent despite our introductory video that discussed our rationale for collecting samples and information, processes for researchers to use samples and information, and plan to ensure the privacy of biobank participants. Lack of trust in researchers and organizing institutions is one possible explanation for discomfort with sharing information and for having common concerns. Improving public trust might then lead to improved comfort with sharing information, reduced occurrences of common concerns, and subsequently improved participation in the biobank. Nobile and colleagues, for example, suggest that trust probably explains why active participants consider risks of participating in studies to be low or nonexistent [29].

In this study, trust in the University of Maryland and trust in medical researchers were both associated with willingness to join the UMBiobank. These findings are similar to those of studies of actual biobank participants, indicating that donors appear to trust researchers and organizing institutions [29]. Lack of trust is one of the greatest barriers inhibiting research participation [30] and may be a particularly important attitudinal barrier to research with African Americans [31]. Building trust therefore is paramount to biobank participation, and becomes more challenging for genetic research as it becomes more distinct from the participant. Researchers conducting genome-wide association studies (GWAS), for example, often utilize anonymized datasets that are removed from usual human subjects review [32], but this consequently can limit the ability to oversee downstream uses of those data [33]. Our findings, however, support a need for the chains of trust to be built through the entire research process. Mechanisms for building trust described by Horn *et al.* [34] include “developing relationships and demonstrating a track record that shows accountability, shared interests, and a concern for the best interests of others.”

### 4.2. Public Perceptions of Health Benefits to Participating in a Biobank

We surveyed respondents about three main categories of health benefit: personal health benefit, familial health benefit, and health benefit for others. We found no influence of personal or familial health benefit on willingness to join the biobank (see Table 4). We did find, however that the majority of respondents agreed it was important that their blood sample be used in research that could improve their own health (64%), and improve the health of people they love (70%). The importance of personal and familial benefit was evident despite the explanation in our introductory video that results of research done on biobank samples would not be returned to individual participants. Assuming that respondents understood the video and interpreted questions about personal and familial health benefit as “direct” benefit through the return of results, however, our findings may be interpreted as “hope” to receive information. This supports findings that research participants often feel that participation in a biobank should be mutually beneficial and express interest in receiving personally relevant clinically significant results (*i.e.*, actionable results) [35,36,37,38]. Returning such results, however, also have the potential to do harm for reasons such as difficulties in understanding genetic risk information [39], the uncertain actionability of some genetic research findings, and the radical actions some participants may take with genetic research findings due to their anxieties and fears. A phenomenon known as ‘therapeutic misconception’, in which participants confuse research with healthcare, could also occur if individual research results are disclosed to participants [40]. Some guidelines advise against the disclosure of results of uncertain significance in genetic studies, partly due to the lack of available resources (genetic counselor, *etc.*) necessary to explain such results to study participants [41]. Additional research is needed to better understand whether there were any misconceptions about the benefits of participating from watching the informational video, or if there was a misunderstanding of the survey questions. Furthermore, it is imperative that we understand the kinds of resources that would be needed to mitigate potential harms of returning research results to biobank participants.

Related to perceived health benefits for others, the majority of respondents agreed it was important that their blood sample be used in research that could improve the health of others of the same race or ethnicity (68%), and improve the health of others in general (73%). In addition, perceived health benefit for others was not as important to respondents who were unsure or not willing to join, compared to respondents who were willing to join (see Table 4). One way to interpret perceived health benefits of others is as reflective of altruism, described by Nobile and colleagues as “a voluntarily performed behavior that intentionally benefits another person without expectation of reward.” Our findings would then indicate that altruistic reasons may not be as important for populations who were unsure or would not be willing to join the biobank compared to those indicating they would be willing to join. However, the idea of altruism has been discussed elsewhere as often having some form of reciprocity [29,42,43,44].

### 4.3. Potential Approaches to Improve Biobank Participation

For respondents indicating they were unsure about joining the UMBiobank, many would be more willing to join with approaches to engage community members and build trust in biobanks that we propose. In ranked order (most indicating a perceived responsiveness to fewest indicating a perceived responsiveness), 68% would be more willing to join the UMBiobank with chances to learn more about the biobank, 50% with chances to be updated about research findings based on biobank samples, 41% by putting community concerns first, 36% with awareness of community members leading biobank research, and 29% with community members having a role in decisions about the biobank (see Table 5). In order to improve public attitudes toward biobanks, these approaches have the potential to build trust while also recognizing limitations to overseeing downstream data use with anonymization, given none require individual-level data. With the instantiation of biobank-linked electronic health records there may be opportunities for other approaches that also utilize individual-level data through more robust data access models that facilitate secure access to biobank data for different purposes (e.g., returning incidental findings to individuals, providing access to anonymized data for genome-wide association study [GWAS] analyses). Given uncertainties about whether participants can be truly informed in the process of opting into unknown future research [45,46], however, we are now beginning to see data access models for removing the need for anonymized data for secondary uses all together with technology-supported approaches to obtain consent as the research is being planned [47,48,49]. Our findings suggest that providing potential participants chances to learn more about the biobank (e.g., educational material) and chances to be updated regularly about the biobank (e.g., press releases, website updates) may be effective approaches to promote the awareness of benefits of biobank participation. Providing opportunities to learn research results through such approaches may help strengthen relationships between investigators and participants [38], and arguably provide personal educational benefit.

### 4.4. Limitations

Our study has some limitations that should be noted. First, our recruitment from two outpatient practices may limit the generalizability of our results given these practices may not be representative of all individuals who could potentially participate in the UMBiobank. Our sample does however have a demographic distribution that is similar to FPI more broadly. Second, we had a 51% response rate. Some non-response could be related to logistical reasons, but it is also possible that non-respondents may be more or less likely to participate in a biobank than those who did respond to the survey. Third, we administered an anonymous survey about a hypothetical biobank. There may be some differences in responses if study participants were instead being asked to join an actual biobank.

## 5. Conclusions

This study identified several potentially mutable factors that influence decisions to join a hypothetical biobank. We also demonstrate our method of prioritizing ways to improve attitudes and opinions about joining a biobank according to perceived responsiveness. We assessed perceived responsiveness to approaches including: chances to learn more about the biobank, chances to be updated regularly about the biobank, prioritizing community concerns, including awareness of community members leading biobank research, and community members having a role in decisions about the biobank. These approaches have the potential to build trust, including providing a venue to address common concerns about biobanks and concerns regarding comfort with sharing samples and information. Tailoring community engagement approaches to the specific concerns of the community in which recruitment will occur and providing regular updates would also promote awareness of benefits to others while also providing some personal benefit though facilitating educational opportunities.

## Supplementary Files

Supplementary File 1
