# Supplementary material for: Prioritizing Approaches to Engage Community Members and Build Trust in Biobanks: A Survey of Attitudes and Opinions of Adults within Outpatient Practices at the University of Maryland
Source: J Pers Med. 2015 Jul 28;5(3):264–79. doi: 10.3390/jpm5030264 (PMC4600147; doi:10.3390/jpm5030264)
Supplement: Supplementary File 1 [file jpm-05-00264-s001.pdf]

Supplementary Material UMBiobank Survey

Q1–3

	Yes	No	Unsure
1. Do you feel like you understand what you just heard?	❍	❍	❍
2. Do you think you would be willing to join the UMBiobank?	❍	❍	❍
3. Would you want University of Maryland deciding how your blood samples and clinical information are used in research?	❍	❍	❍

Q4-11 Please show how comfortable you are with the UMBiobank using your samples and different kinds of information:

	Comfortable with the UMBiobank using	Neither comfortable nor uncomfortable with the UMBiobank using	Uncomfortable with the UMBiobank using
4. Your blood samples	❍	❍	❍
5. Your age	❍	❍	❍
6. Your gender	❍	❍	❍
7. Your ethnic group	❍	❍	❍
8. Your previous illnesses and diagnoses	❍	❍	❍
9. Your test results (e.g., any lab results, x-rays)	❍	❍	❍
10. Your previous treatments (e.g., medications)	❍	❍	❍
11. Your genetic information or genetic test results	❍	❍	❍

Q12-16 Please show how your willingness to join the UMBiobank would change if:

	I would be more willing to join the UMBiobank	No change	I would be less willing to join the UMBiobank
12. Members of my community have a role in making decisions about the biobank.	❍	❍	❍
13. There are chances to be updated regularly about the biobank (e.g., press releases, website updates).	❍	❍	❍
14. There are chances to learn more about the biobank (e.g., educational material).	❍	❍	❍
15. Concerns of my community are put first.	❍	❍	❍
16. Members of my community are leading biobank research.	❍	❍	❍

Q17-21 Some people may have concerns about participating in a biobank. Please indicate your level of concern with the following:

	Concerned	Neither concerned nor unconcerned	Not concerned
17. Researchers having my samples and information.	❍	❍	❍
18. Keeping my information private.	❍	❍	❍
19. Information stored in the biobank being used against me.	❍	❍	❍
20. Feeling like a guinea pig.	❍	❍	❍
21. Information stored in the biobank being used to discriminate against people by race or ethnicity.	❍	❍	❍

Q22-29 Please show if you agree or disagree with the following statements:

	Agree	Neither agree nor disagree	Disagree
22. It is important that my blood sample be used in research that could improve my own health.	❍	❍	❍
23. It is important that my blood sample be used in research that will not affect my own health, but could improve the health of people I love.	❍	❍	❍
24. It is important that my blood sample be used in research that will not affect my own health, or the health of people I love, but could improve the health of others of the same race or ethnicity.	❍	❍	❍
25. It is important that my blood sample be used in research that will not affect my own health, or the health of people I love, but could improve the health of others in general.	❍	❍	❍
26. Medical researchers care only about what is best for each patient.	❍	❍	❍
27. Medical researchers tell patients everything they need to know about being in a research study.	❍	❍	❍
28. Medical researchers treat people like "guinea pigs."	❍	❍	❍
29. I completely trust doctors who do medical research.	❍	❍	❍

Please answer the following questions about your background:

Q30 What is your gender?

- ❍ Male
- ❍ Female
- ❍ Other
- ❍ Prefer not to say

Q31 What is your ethnic background?

- ❍ Hispanic or Latino
- ❍ Non-Hispanic
- ❍ Prefer not to say

Q32 What is your race?

- ❍ Black or African American
- ❍ White or Caucasian
- ❍ American Indian or Alaska Native
- ❍ Hawaiian or Pacific Islander
- ❍ Asian or Asian American
- ❍ Prefer not to say

Q33 How old are you?

- ❍ 18–29 years old
- ❍ 30–44 years old
- ❍ 45–59 years old
- ❍ >60 years old

Q34 What is your highest level of education?

- ❍ Less than high school
- ❍ High school graduate or GED
- ❍ Some college
- ❍ Bachelor’s degree
- ❍ Graduate or professional degree

Q35 Do you have children (biological and/or adopted/step/foster)?

- ❍ Yes
- ❍ No

Q36 Have you ever donated blood?

- ❍ Yes
- ❍ No
- ❍ I tried, but was not accepted

Q37 Have any of your 1st degree relatives (parents, siblings, children) been affected by a major illness?

- ❍ Yes
- ❍ No
- ❍ Unsure

© 2015 by the authors; licensee MDPI, Basel, Switzerland. This article is an open access article distributed under the terms and conditions of the Creative Commons Attribution license (http://creativecommons.org/licenses/by/4.0/).
